# A Single Point Mutation Blocks the Entrance of Ligands to the Cannabinoid CB_2_ Receptor via the Lipid Bilayer

**DOI:** 10.1021/acs.jcim.2c00865

**Published:** 2022-10-27

**Authors:** Nil Casajuana-Martin, Gemma Navarro, Angel Gonzalez, Claudia Llinas del Torrent, Marc Gómez-Autet, Aleix Quintana García, Rafael Franco, Leonardo Pardo

**Affiliations:** † Laboratory of Computational Medicine, Biostatistics Unit, Faculty of Medicine, Universitat Autònoma Barcelona, 08193 Bellaterra, Barcelona, Spain; ‡ Department of Biochemistry and Physiology, Faculty of Pharmacy and Food Sciences, Universitat de Barcelona, 08028 Barcelona, Spain; § Centro de Investigación en Red, Enfermedades Neurodegenerativas (CIBERNED), Instituto de Salud Carlos III, 28031 Madrid, Spain; ∥ Department of Biochemistry and Molecular Biomedicine, Faculty of Biology, Universitat de Barcelona, 08028 Barcelona, Spain

## Abstract

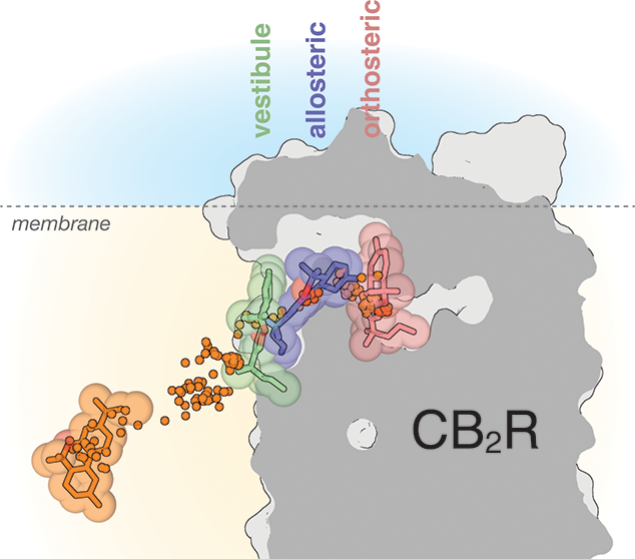

Molecular dynamic (MD) simulations have become a common tool to study the pathway of ligand entry to the orthosteric binding site of G protein-coupled receptors. Here, we have combined MD simulations and site-directed mutagenesis to study the binding process of the potent JWH-133 agonist to the cannabinoid CB_2_ receptor (CB_2_R). In CB_2_R, the N-terminus and extracellular loop 2 fold over the ligand binding pocket, blocking access to the binding cavity from the extracellular environment. We, thus, hypothesized that the binding pathway is a multistage process consisting of the hydrophobic ligand diffusing in the lipid bilayer to contact a lipid-facing vestibule, from which the ligand enters an allosteric site inside the transmembrane bundle through a tunnel formed between TMs 1 and 7 and finally moving from the allosteric to the orthosteric binding cavity. This pathway was experimentally validated by the Ala282^7.36^Phe mutation that blocks the entrance of the ligand, as JWH-133 was not able to decrease the forskolin-induced cAMP levels in cells expressing the mutant receptor. This proposed ligand entry pathway defines transient binding sites that are potential cavities for the design of synthetic modulators.

## 1 Introduction

The approximately 800 (∼450 for sensory and ∼350 for nonsensory functions) members of the G protein-coupled receptor (GPCR) family are mainly formed by a conserved architecture of seven transmembrane domains (TMs).^1^ The intracellular domains and/or the intracellular part of the TM segments bind a small repertoire of signaling proteins (16 G proteins, four arrestins, or seven G protein-coupled receptor kinases),^2−4^ whereas the extracellular domains (ECDs) and/or the extracellular part of the TM segments recognize exogenous signals (e.g., 1 trillion olfactory^5^ or ∼1 000 bitter molecules^6^), or endogenous signals that range in size from ions (e.g., Ca^2+^) to small molecules (e.g., glutamate, neurotransmitters) to peptides (e.g., opioids, endothelin, glucagon) or large proteins (e.g., chemokines, glycoprotein hormones), or ∼200 000 synthetic ligands,^7^ or ∼475 drugs.^8^ Thus, the mechanism of binding of this set of highly diverse extracellular ligands varies considerably depending on the ligand and receptor,^9^ as subsequently confirmed by the analysis of known inactive and active structures of GPCRs.^10^

The high druggability of membrane embedded GPCRs^11^ is due to the fact that their malfunction, with 435 disease-associated mutations,^12^ commonly translates into pathological outcomes^13^ and because the orthosteric binding site, a conserved pocket within the 7TM domain that optimally accommodates the electrostatic and steric properties of the ligand, is normally accessible from the extracellular space. However, the depth of ligand penetration into the TM bundle is considerable,^14^ so ligands must also engage stable interactions at the entrance of the binding site. Ligand binding pathway simulations on β-adrenergic receptors have identified these additional cavities that are transiently occupied before arrival to the orthosteric binding site.^15,16^ They have been named extracellular vestibules^15^ or entrances,^17^ or secondary^16,18^ or metastable^19^ binding sites, or exosites.^20^ Cavities like those described for β-adrenergic receptors have also been found for the M3 muscarinic receptor,^21^ the adenosine A_2A_ receptor,^22^ the histamine H_4_ receptor,^23^ the dopamine D_2_ and D_3_ receptors,^24^ and olfactory receptors,^25^ among others. Remarkably, while the orthosteric binding site is conserved among family members, these sites at the ECDs are highly divergent and have become potential binding sites for synthetic modulators.^26^ Thus, they have been used in the design of allosteric modulators^27^ or bitopic ligands,^20,28^ as well as proposed to bind short bivalent ligands.^29,30^

Class A GPCRs activated by hormone-like signaling molecules derived from lipid species with long hydrophobic moieties^31^ possess a distinctive structural signature at the ECD, in comparison with other class A GPCRs that are activated by polar ligands.^32^ In the crystal structures of S1P_1_,^33^ LPA1,^34^ FFAR1,^35^ CRTH2,^36^ CB_1_,^37,38^ and CB_2_^39^ receptors, the extracellular N-terminus and extracellular loop 2 fold over the ligand binding pocket. Thus, the entrance of ligands to the orthosteric binding site through the membrane bilayer has been proposed for S1P_1_,^33,40^ CRTH2,^36^ and CB_1_^37,41^ receptors via TMs 1 and 7, for the FFAR1 via TMs 3 and 4,^35^ for the MT1 melatonin receptor via TMs 4 and 5,^42^ for opsin via TMs 5 and 6,^43,44^ and for CB_2_^45^ and PAR1^46^ receptors via TMs 6 and 7. In contrast, some authors propose that hydrophobic ligands of LPA1^34^ and CB_1_ and CB_2_^47^ receptors may reach the orthosteric site from the extracellular environment.

In this manuscript, we combine molecular dynamic (MD) simulations and site-directed mutagenesis to study the binding process of a potent and selective CB_2_R agonist, JWH-133. The proposed binding pathway is a multistage process consisting of the ligand diffusing in the lipid bilayer to contact a lipid-facing vestibule, from which the ligand enters an allosteric site inside the TM bundle through a tunnel formed between TMs 1 and 7, and finally moving from the allosteric to the orthosteric binding cavity.

## 2 Materials and Methods

### 2.1 Initial CB_2_R Models

The inactive structure of CB_2_R was used (PDB id 5ZTY).^39^ Protonation states were assigned with PROPKA.^48^ The system was oriented by the Orientations of Proteins in Membranes (OPM) database^49^ and embedded in four different lipid bilayer boxes (9 × 9 × 9.5 nm), constructed using PACKMOL-memgen,^50^ containing 1-palmitoyl-2-oleoyl-*sn*-glycero-3-phosphocholine (POPC), cholesterol (CHL; 10:6 POPC/CHL ratio), water molecules (TIP3P), and monatomic Na^+^ and Cl^–^ ions (0.15 M). JWH-133 (JWH) was inserted into the membrane by replacing one in six CHL molecules from both leaflets, leading to a 10:5:1 POPC/CHL/JWH ratio. This substitution of CHL by JWH was randomly performed five times for each of the four constructed membrane systems (see Figure S1).

### 2.2 Molecular Dynamics Simulations

These 20 combinations of CB_2_R models were simulated with GROMACS2016.4.^51^ The amber14sb-ildn force field was used for the protein, solvent. and ions;^52^ a GROMACS adaptation of lipid14 for lipids;^53,54^ and the general Amber force field (GAFF2) with HF/6-31G*-derived RESP atomic charges for JWH-133.^55^ Molecular systems were subjected to 10 000 steps of energy minimization, followed by 20 ns of gradual relaxation of positional restraints (corresponding to 100, 50, 25, and 10 kJ mol^–1^ nm^–2^) at protein backbone coordinates before the production phase to hydrate the cavities and allow lipids to pack around the protein. After equilibration, five replicas of 1 μs unrestrained MD trajectory, of each of the 20 combinations, was generated at a constant temperature of 300 K using separate v-rescale thermostats for the receptor, ligand, lipids, and solvent molecules. A time step of 2.0 fs was used for the integration of equations of motions. Bonds involving hydrogen atoms were kept frozen using the LINCS algorithm. Lennard-Jones interactions were computed using a cutoff of 1.1 nm, and the electrostatic interactions were treated using PME with the same real-space cutoff under periodic boundary conditions (see Figure S1). The analysis of the trajectories was performed using MDAnalysis;^56^ the stability of the membrane was monitored (see Figure S2) using FATSLiM.^57^ Visualization and image rendering were performed with PyMOL^58^ and VMD,^59^ and the tunnels of CB_2_R, from the orthosteric binding cavity, were calculated with the CAVER program.^60^

### 2.3 Metadynamics Simulations

**Figure 1:**
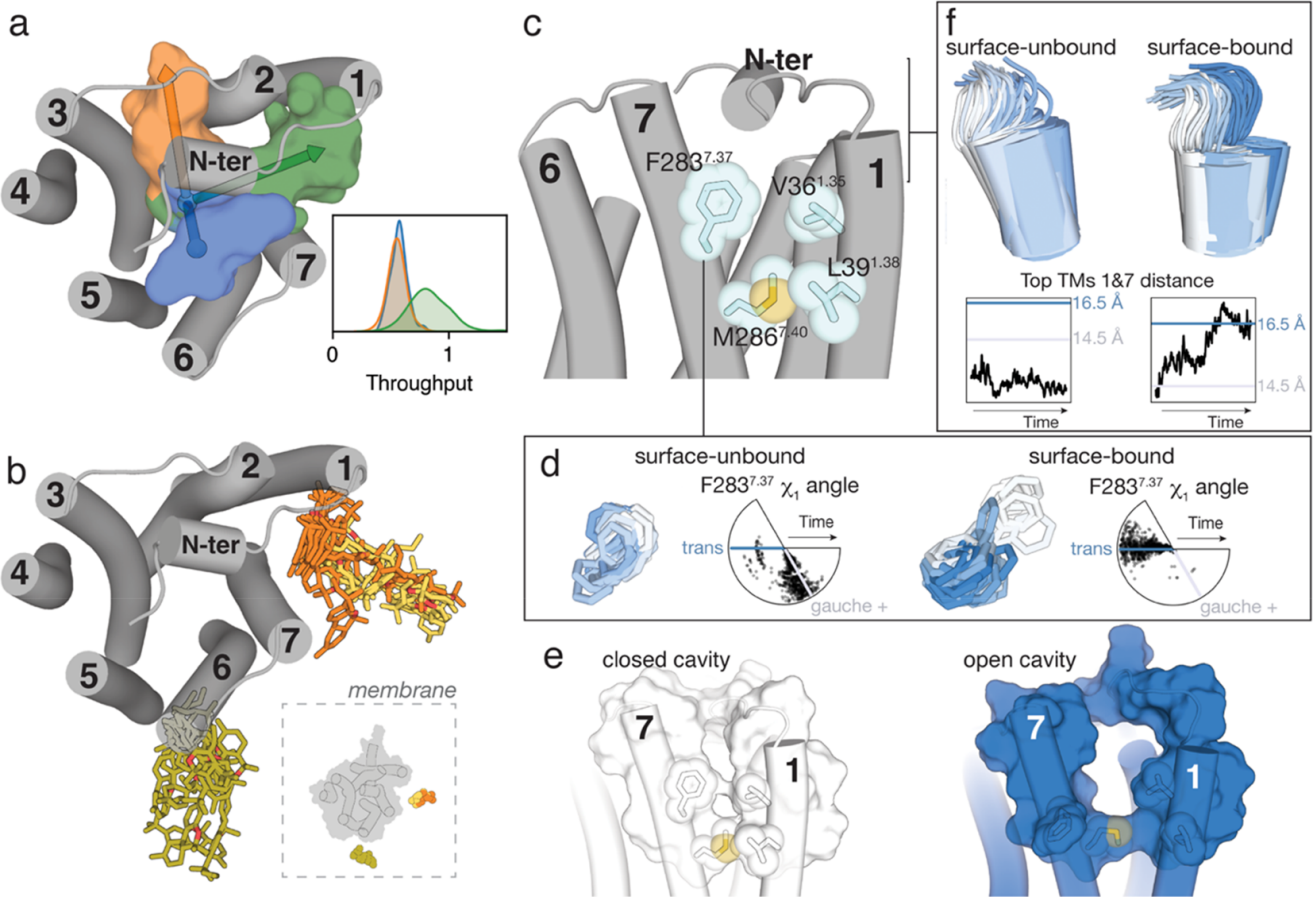
Ligand diffusion to CB_2_R. (a) Cluster of tunnels, as calculated with CAVER,^60^ from the orthosteric cavity toward the extracellular domain (blue) and toward the lipid bilayer via either TMs 2 and 3 (orange) or TMs 1 and 7 (green). The probabilities of ligand transportation (throughput) of each tunnel, calculated from the ensemble of structures collected during the aggregated 100 μs of MD simulation, are shown. (b) In three of the 100 MD simulations, the ligand diffused from the lipid bilayer to the receptor surface (10 snapshots per simulation are shown) and remained bound to either TMs 5 and 6 (one simulation in green) or TMs 1 and 7 (two simulations in orange and yellow). (c–f) Comparison of the trajectories (Figure S3) in which the ligand spontaneously bound the lipid-facing part of TMs 1 and 7 (orange in panel b, surface-bound) and a trajectory in which the ligand did not bind the receptor surface (surface-unbound). Detailed view of key amino acids located at the entrance of the TMs 1 and 7 tunnel (c). Evolution of the Phe283^7.37^ side chain and the χ_1_ dihedral angle along the surface-unbound (left) and surface-bound (right) trajectories (d). Representative structures obtained during the MD simulations (Figure S3) in which the cavity is closed (in white) or open (in blue). The conformations of Phe283^7.37^ in panel d and TM 1 in panel f that resemble these closed or open structures are shown in white or blue, respectively. Evolution of TM 1 and the distance between the top of TMs 1 (Cα atom of Thr34^1.33^) and 7 (Cα atom of Val277^7.31^) along the surface-unbound (left) and surface-bound (right) trajectories (f).

Metadynamics simulations (see Figure S1) were performed using GROMACS2016.4 patched with PLUMED version 2.5^61^.^62^ The starting point was taken from one of the 100 unbiased MD trajectories in which JWH-133 spontaneously bound the lipid-facing part of TMs 1 and 7 (orange in Figure 1b). The end point is the docking pose of JWH-133 to the orthosteric binding site of CB_2_R previously reported,^63^ using the structurally similar AM12033 ligand (PDB id 6KPC)^47^ as a template. The distance between the center of mass at the starting and end points of JWH-133 was used as a collective variable (CV). This CV was selected for simplicity and because it can efficiently represent the dimensional space of the free-energy of binding. In metadynamics simulations, a history-dependent potential is applied along the CV, built as a sum of Gaussian kernels, with a specific width of 0.1, height of 0.48 kJ/mol, and pace of 5000 steps (i.e., 0.1 ns). A light RMSD constraint (with an energy constant KAPPA of 200 IU) was applied to ensure a correct end conformation of JWH-133 at the orthosteric site. We used well-tempered metadynamics, where the height of the applied Gaussian potentials is rescaled so that the system does not explore free-energy regions beyond the expected values. Thus, a bias factor of 20, accounting for the difference between the system temperature and the temperature of the CV, was set up. Convergence of the well-tempered metadynamics simulations was determined when JWH-133 had explored all of the CV space and the free-energy profile was constant at 10 ns intervals. Free-energy profiles were calculated using PLUMED sum_hills function.

### 2.4 CB_2_R Mutants

Mutations were produced using the QuikChange Site-Directed Mutagenesis Kit. The cDNA for hCB_2_R, cloned into pcDNA3.1, was amplified using sense and antisense primers harboring the triplets for the desired point mutation (Pfu turbo polymerase was used). The forward and reverse primers are ccccagaagacagctTTTgctgtgttgtgcactc and gagtgcacaacacagcAAAagctgtcttctgggg for Val36^1.35^Phe, ccccagaagacagctATGgctgtgttgtgcactc and gagtgcacaacacagcCATagctgtcttctgggg for Val36^1.35^ Met, gtcaagaaggcctttTTCttctgctccatgctg and cagcatggagcagaaGAAaaaggccttcttgac for Ala282^7.36^Phe, and gtcaagaaggcctttATGtctgctccatgctg and cagcatggagcagaaCATaaaggccttcttgac for Ala282^7.36^Phe, respectively, in which mutated nucleotides are written in upper case characters. The nonmutated DNA template was digested for 1 h with DpnI. PCR products were used to transform XL1-blue supercompetent cells. Finally, positive colonies were tested by sequencing to select those expressing the correct DNA sequence.

### 2.5 cAMP Determination Assays

Determination of cAMP levels in HEK-293T cells transiently expressing CB_2_R (1 μg of cDNA) or the mutant version of the receptor was performed using the Lance-Ultra cAMP kit (PerkinElmer). Two hours before initiating the experiment, the medium was substituted by a serum-free medium. Then, transfected cells were dispensed in white 384-well microplates at a density of 3000 cells per well and incubated for 15 min at room temperature with compounds, followed by 15 min of incubation with forskolin and 1 h more with homogeneous time-resolved fluorescence (HTRF) assay reagents. Fluorescence at 665 nm was analyzed on a PHERAstar Flagship microplate reader equipped with an HTRF optical module (BMG Labtech). Data analysis was made based on the fluorescence ratio emitted by the labeled cAMP probe (665 nm) over the light emitted by the europium cryptate-labeled anti-cAMP antibody (620 nm). A standard curve was used to calculate cAMP concentration. Forskolin-stimulated cAMP levels were normalized to 100%.

## 3 Results

### 3.1 Computational Model to Study the Pathway of Ligand Entry to CB_2_R

The selected ligand is JWH-133, a potent selective CB_2_R agonist.^64^ We have chosen JWH-133 over other agonists for its rigidity and for having only one heteroatom in its structure. JWH-133 (6.4) and cholesterol (8.7) have similar computed logP values, which makes JWH-133 a potential through-the-membrane binder. The model to study the entry of JWH-133 into the orthosteric binding site consisted of inactive CB_2_R embedded in a lipid bilayer box (see Materials and Methods). Four different membranes were constructed, and for each of them, JWH-133 was positioned at five different positions. These 20 combinations were each subjected to five rounds of unbiased 1 μs MD simulation with an aggregate sampling of 100 μs (see Methods and Figure S1). Data was collected every 10 ns.

### 3.2 Tunnels in CB_2_R

To delineate the pathways from the orthosteric binding cavity of CB_2_R, buried in the TM bundle, to the surrounding solvent, either the aqueous extracellular environment or the lipid bilayer, we used the ensemble of structures collected during the MD simulations of CB_2_R. Figure 1a displays three different clusters of tunnels calculated with the skeleton search algorithms implemented in the CAVER program.^60^ These are toward the extracellular domain and toward the lipid bilayer via either TMs 2 and 3 or TMs 1 and 7. The tunnel route preference was evaluated using *throughput*, which is the probability of ligand transportation throughout the pathway (the higher the value, the greater the importance of the pathway). These values of *throughput* (Figure 1a), which consider the conformational flexibility of CB_2_R, suggest that the transit of molecules through the lipidic phase via TMs 1 and 7 is favored compared to TMs 2 and 3 or to the extracellular route.

### 3.3 Ligand Diffusion to CB_2_R and Tunnel Opening

None of the JWH-133 agonists, embedded at different positions in the membrane, spontaneously bound the CB_2_R orthosteric binding site during the aggregated 100 μs of MD simulation, but in three simulations the ligand remained bound to the receptor surface, mainly to amino acids in TMs 5 and 6 (one simulation) or TMs 1 and 7 (two simulations). Figure 1b shows these stable surface-bound ligand conformations with root-mean-square deviation (rmsd) values < 20 Å relative to the reference docked binding mode of JWH-133 (see Materials and Methods), while they have displaced >20 Å from their initial position. These results, together with the *throughput* values of tunnel route preference (see above), reinforce TMs 1 and 7 as the most feasible pathway of ligand entry to the orthosteric site of CB_2_R. Figure 1c shows the key Val36^1.35^, Leu39^1.38^, Phe283^7.37^, and Met286^7.40^ residues (Ballesteros and Weinstein numbering scheme^65^ as superscript) located at the entrance of TMs 1 and 7. Of note is the conformation of Phe283^7.37^, which in the *gauche+* conformation, like that observed in the crystal structures of CB_2_R,^39,47,66^ closes the tunnel, whereas in the *trans* conformation, it opens it. Moreover, it has been shown that the N-terminus of the S1P_1_ receptor packs against ECL-2 in the active conformation, leading to an opening of the ligand access vestibule between TMs 1 and 7.^67^ Thus, we have quantified in Figure S3 the number of snapshots, during the 100 μs of MD simulation, in which Phe283^7.37^ adopts the *trans* conformation or/and the distance between the top of TMs 1 (Cα atom of Thr34^1.33^) and 7 (Cα atom of Val277^7.31^) increases from the initial value of 14.5 Å to values larger than 15.5 Å. GPCRs are dynamic proteins that permit rapid small-scale structural fluctuations,^68^ and accordingly, both events can be freely observed, simultaneously or not, during the calculated trajectories (Figure S3). Importantly, the trajectory in which the ligand (orange in Figure 1b) spontaneously binds the lipid-facing part of TMs 1 and 7 (surface-bound, Figure S3) triggers or stabilizes the *trans* conformation of Phe283^7.37^ (Figure 1d), and the distance between TMs 1 and 7 increases to 16.5 Å (Figure 1f) most of the simulation time. In contrast, in other trajectories in which the ligand did not bind the receptor surface and these events did not spontaneously occur (surface-unbound, Figure S3), Phe283^7.37^ remains in the *gauche+* conformation (Figure 1d) and the distance between TMs 1 and 7 decreases to values lower than the initial 14.5 Å (Figure 1f). Figure 1e shows two selected snapshots of these surface-unbound and surface-bound trajectories (see Figure S3) in which the tunnel between TMs 1 and 7 is closed and open, respectively.

### 3.4 Ligand Access to the Orthosteric Binding Site

**Figure 2:**
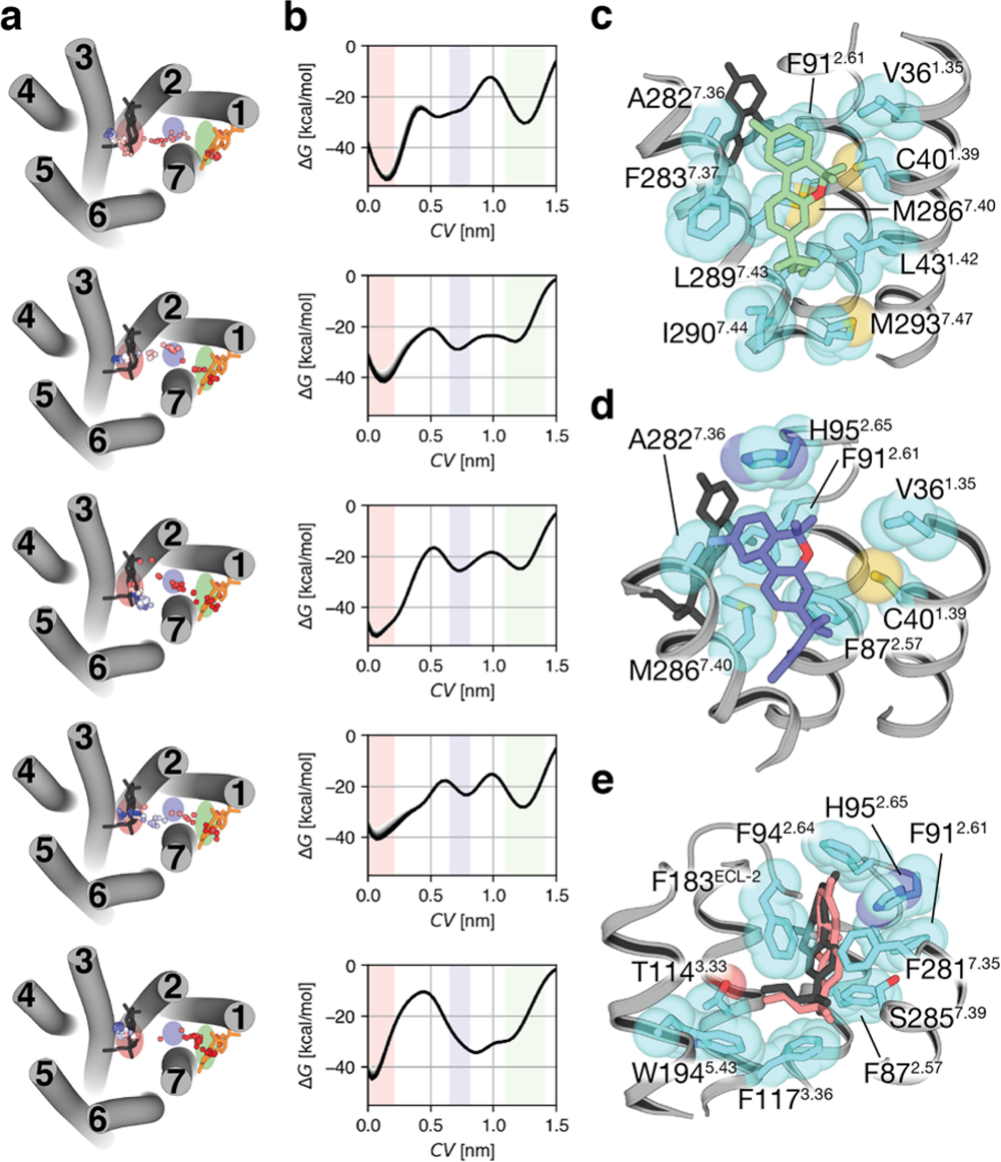
Well-tempered metadynamics simulations of the pathway of ligand entry to the orthosteric binding site of CB_2_R. (a) The ligand pathway from the initial position of JWH-133 (in orange, see Figure 1b) to the final reference position bound to the orthosteric site of CB_2_R (in black), obtained during the well-tempered metadynamics, is represented by dots (the dot color gradient, from red to blue, corresponds to simulation time, from the beginning to the end, respectively). Five independent replicas are shown. (b) The free-energy profile of the five replicas (the last 10 snapshots before convergence are plotted). The collective variable (CV) is the distance between the center of mass of JWH-133 in the initial and final positions. Three energetic minima are observed when the ligand is bound to the lipid-facing part of the tunnel (green rectangle), to the bundle-facing part of the tunnel (blue), or to the orthosteric site (pink). These three positions in the ligand pathway are depicted in panel a by an ellipse with a color matching to the colors in panel b. The initial state of the binding process is on the right part of the graph and the final state on the left part. (c–e) Representative structures of JWH-133 and the interacting side chains, along the ligand pathway, at the lipid-facing part (c), the bundle-facing part (d), and the orthosteric site (e). The color of JWH-133 corresponds to the color of the minima, whereas the final reference position is shown in black.

The above results, and the experimental evidence (see Introduction), led us to use the ligand bound to TMs 1 and 7 of CB_2_R to progressively sample the binding event. Because entry to the orthosteric binding site can have the largest energetic barrier of the process,^15,40^ in some cases unsurmountable by unbiased MD simulations, we used well-tempered metadynamics.^69^ In this technique, a biasing potential is applied to permit the system to explore energetically inaccessible regions for unbiased MD simulations on reasonable time scales. We used as a collective variable of the distance between the center of mass of the ligand in the initial conformation and the reference docking pose at the orthosteric binding site (see Materials and Methods). In the free-energy profile of five independent simulations (Figure 2a and b), three minima can be noticed in most trajectories. These steps in the entry pathway were designated as lipid-facing, bundle-facing, and orthosteric. In the first minimum, the ligand is bound to the lipid-facing part of the CB_2_R tunnel mainly formed by amino acids (Val36^1.35^, Cys40^1.39^, Leu43^1.42^, Ala282^7.36^, Phe283^7.37^, Met286^7.40^, Leu289^7.43^, Ile290^7.44^, and Met293^7.47^) in TMs 1 and 7 (Figure 2c). In the second minimum, the ligand is bound to the bundle-facing part of the CB_2_R tunnel mainly formed by amino acids (Val36^1.35^, Cys40^1.39^, Phe87^2.57^, Phe91^2.61^, His95^2.65^, Ala282^7.36^, and Met286^7.40^) in TMs 1, 2, and 7 (Figure 2d). In the final third minimum of the binding process, JWH-133 reaches the orthosteric binding site. This computationally derived binding pose, using metadynamics, reproduced the main contacts between JWH-133 and the receptor (Phe87^2.57^, Phe91^2.61^, Phe94^2.64^, His95^2.65^, Thr114^3.33^, Phe117^3.36^, Phe183^ECL2^, Trp194^5.43^, Phe281^7.35^, and Ser285^7.39^) previously proposed^63^ (Figure 2e).

### 3.5 Experimental Validation of the Pathway of Ligand Entry

**Figure 3:**
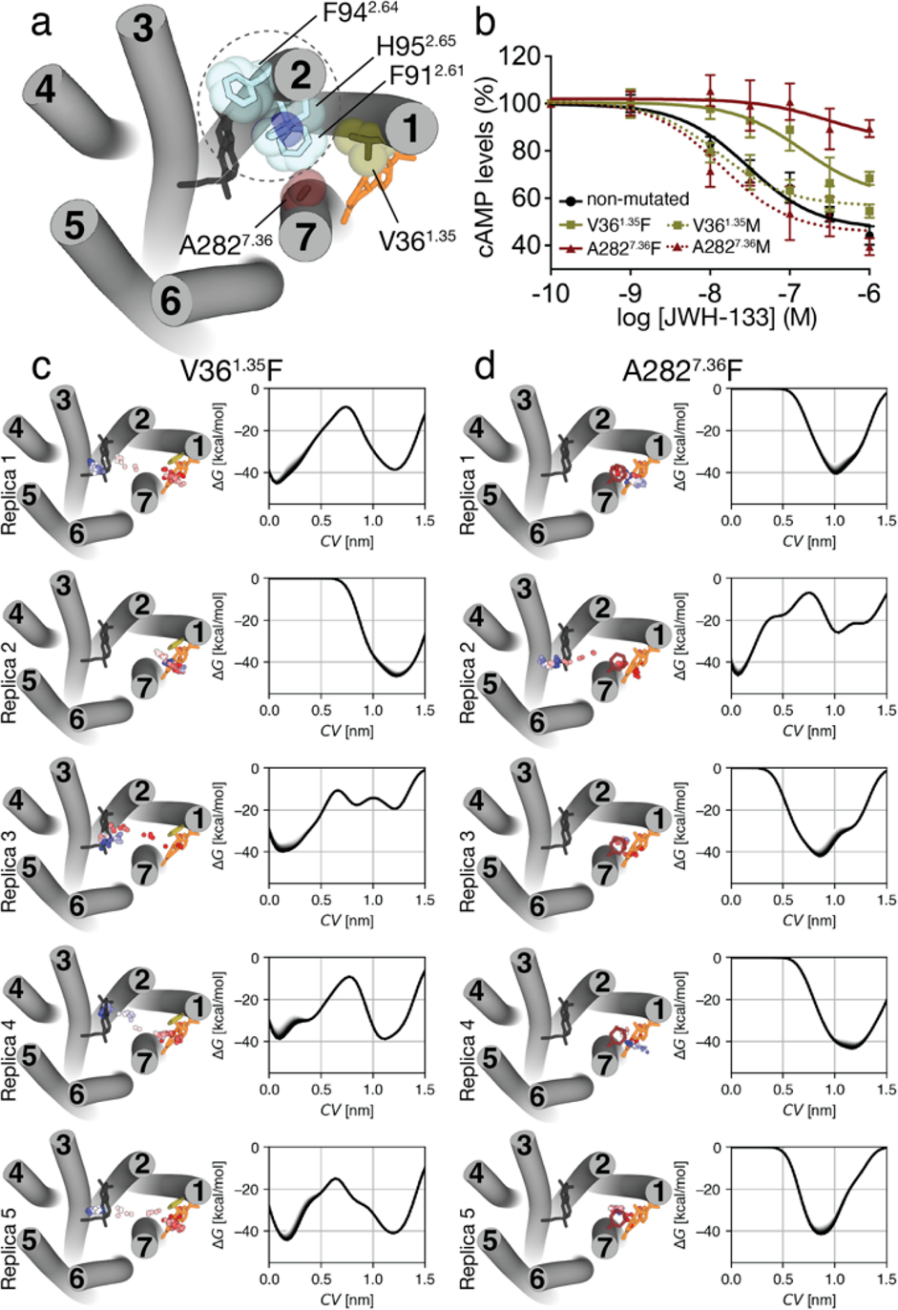
A single point mutation blocks the entrance of the ligand. (a) Extracellular view of CB_2_R (ECLs are omitted for clarity) in which the position of Val36^1.35^ (green surface) and Ala282^7.36^ (red), located between the lipid-facing (Figure 2c) and bundle-facing (Figure 2d) minima, and Phe91^2.61^, Phe94^2.64^, and His95^2.65^ side chains in TM 2 (blue) are shown. (b) Decrease of forskolin-induced cAMP (normalized to 100%), in HEK-293T cells, upon stimulation of nonmutated (black line), Val36^1.35^Phe (green) and Ala282^7.36^Phe (red) mutations (mutation to Phe is shown as a solid line), and Val36^1.35^ Met (green) and Ala282^7.36^ Met (red) mutations (mutation to Met is shown as a dotted line) with the JWH-133 agonist. (c,d) Ligand pathways and free-energy profiles (the last 10 snapshots before convergence are plotted) of five replicas, obtained in well-tempered metadynamics of JWH-133 entry to the Val36^1.35^Phe (c) or Ala282^7.36^Phe (d) mutant receptors. The collective variable (CV) is the distance between the center of mass of JWH-133 in the initial (orange) and final (black) conformations. See legend of Figure 2 for additional details.

To experimentally validate the proposed mechanism of ligand entry by site-directed mutagenesis, we explored the amino acids involved in the transition from the lipid- to the bundle-facing part of the CB_2_R tunnel (Figure 2c,d). Val36^1.35^ and Ala282^7.36^ are located midway between these two minima, delimiting the tunnel between TMs 1 and 7. Notably, three aromatic, Phe91^2.61^, Phe94^2.64^, and His95^2.65^, side chains in TM 2 are in their environment (Figure 3a). Thus, we obtained the Val36^1.35^Phe and Ala282^7.36^Phe mutant versions of the CB_2_R with the main idea that the aromatic side chains would form a stable aromatic cluster with Phe91^2.61^. Phe94^2.64^, and/or His95^2.65^, blocking the access of the ligand to the orthosteric binding site. Dose–response experiments in HEK-293T cells expressing the CB_2_R stimulated with forskolin and treated with JWH-133 showed reduced cAMP production (Figure 3b and Table 1), as expected for G_i_-coupled receptors. Clearly, both Val36^1.35^Phe and Ala282^7.36^Phe mutations impair the effect of JWH-133, as the decrease of forskolin-induced cAMP is of less magnitude in the mutant receptors (the effect is more noticeable in Ala282^7.36^Phe than in Val36^1.35^Phe).

**Table 1:** Functional Properties of JWH-133 at Nonmutated and Mutated CB_2_R

	pEC_50_^a^	*E*_max_^b^
nonmutated	7.5 ± 0.1	47.0 ± 3.5
Val36^1.35^Phe	6.8 ± 0.2	60.2 ± 6.7
Ala282^7.36^Phe	6.5 ± 0.6	84.3 ± 8.4
Val36^1.35^ Met	7.9 ± 0.1	56.7 ± 1.7
Ala282^7.36^ Met	7.8 ± 0.2	45.4 ± 4.2

a pEC_50_ (nM).

b *E*_max_ (%), the maximum inhibition of forskolin-stimulated cAMP levels (normalized to 100%). These values were calculated using nonlinear regression analysis. Data are expressed as the mean ± SE of at least nine independent experiments performed in triplicate.

The importance of these stable aromatic interactions was tested by comparing the results with those obtained using Val36^1.35^ Met and Ala282^7.36^ Met mutants (Figure 3b and Table 1). The highly polarizable sulfur atom of the Met side chain can form with other aromatic side chains stronger interactions than Phe.^70,71^ Interestingly, these new mutations do not affect the decrease in forskolin-induced cAMP in a significant manner, thus suggesting they do not block the entrance of the JWH-133 ligand probably due to the higher flexibility of Met relative to Phe. Our experimental results point out that the highly stable aromatic cluster between Phe91^2.61^, Phe94^2.64^, His95^2.65^, and Phe282^7.36^ (in the Ala282^7.36^Phe mutation) blocks the entrance of JWH-133 to the orthosteric binding site.

### 3.6 Ligand Access to the Binding Site in the Val36^1.35^Phe and Ala282^7.36^Phe Mutant Receptors

To study the influence of this aromatic cluster, attained in the Val36^1.35^Phe and Ala282^7.36^Phe mutants, during the process of ligand entry, we performed well-tempered metadynamics using the same protocol as for nonmutated CB_2_R. As expected, the Val36^1.35^Phe mutation hampers the ability of the ligand to access the orthosteric site in one of the replicates and the possibility to explore the minimum corresponding to the bundle-facing part of the CB_2_R tunnel in three other replicates (Figure 3c). On the other hand, the Ala282^7.36^Phe mutation blocks the entrance of the ligand in four of the five replicates performed (Figure 3d) in agreement with our hypothesis and experimental results.

## 4 Discussion and Conclusions

Drug-target residence time is a key element as a biological effect requires binding to the target receptor.^72,73^ Residence time is related to the ligand–receptor binding kinetics and, thus, to the pathway used in ligand association and dissociation.^74^ MD simulations have become a convenient tool to estimate residence times^75,76^ and to analyze such pathways.^15,16,21−25,40,77^ These simulations show that ligand binding is a multistage process in which the ligand forms key interactions with amino acids at the entrance of the binding site. Most of these simulations are for class A GPCRs that bind polar ligands directly from the extracellular environment.

**Figure 4:**
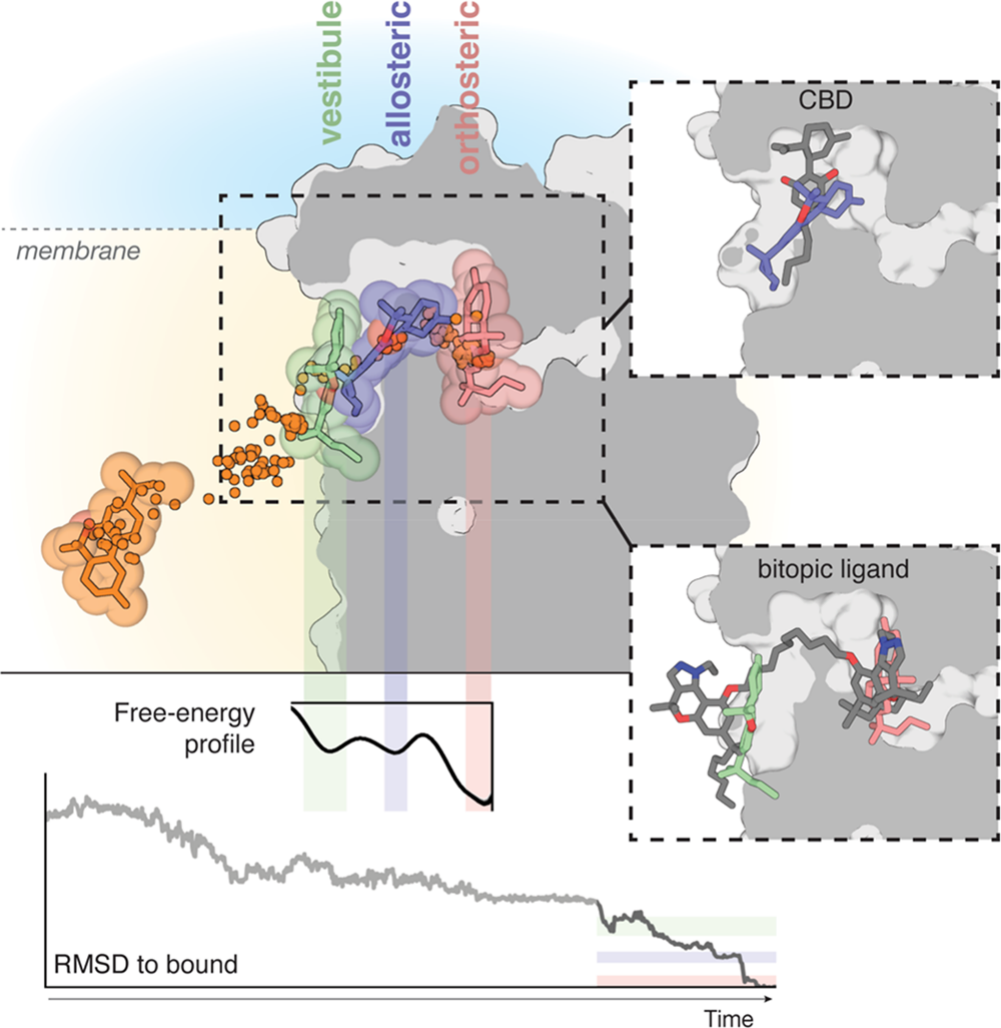
The binding pathway of JWH-133 to the orthosteric site of CB_2_R. JWH-133 (in orange) diffuses through the membrane to reach a minimum in the free-energy profile in which JWH-133 (in green) contacts a membrane vestibule or lipid-facing cavity. Subsequently, JWH-133 (in blue) moves to a second allosteric minimum or bundle-facing cavity in the trajectory. Finally, JWH-133 (in pink) moves to the most stable minimum in the pathway at the orthosteric binding site. Ligand positions during the pathway are shown by dots. RMSD values (light gray from unbiased MD simulations, dark gray from well-tempered metadynamics) relative to the reference docked binding mode of JWH-133 at the orthosteric site and a representative free-energy profile obtained in well-tempered metadynamics are shown. Color bars indicate the different locations of the ligand. Comparison of our previously reported binding mode of the negative allosteric modulator cannabidiol (CBD, gray)^63^ and JWH-133 at the allosteric minimum or bundle-facing cavity (in blue), and the binding mode of a bitopic ligand (compound **22**, gray)^78^ and JWH-133 at the vestibule or lipid-facing cavity (in green) and the orthosteric binding site (in pink).

Here, we have studied the binding pathway of the potent JWH-133 agonist to CB_2_R by MD simulations. In CB_2_R, the N-terminus and ECL-2 that lacks the disulfide bridge to TM 3, characteristic of the GPCR family, fold over the ligand binding pocket^39^ blocking access to the orthosteric binding cavity from the extracellular environment. We, thus, propose that the hydrophobic JWH-133 (calculated logP of 6.4) diffuses through the bilayer leaflet to contact a lipid-facing cavity of CB_2_R mainly formed by amino acids in TMs 1 and 7 (Figure 4). In this point of the pathway, the ligand forms hydrophobic/aromatic contacts with Leu39^1.38^, Leu42^1.41^, Leu43^1.44^, Phe283^7.37^, Met286^7.40^, and Ile290^7.44^ (Figure 2c). Importantly, the binding of the ligand to this lipid-facing cavity triggers and/or stabilizes the *trans* conformation of Phe283^7.37^ (Figure 1d) and increases the distance between TMs 1 and 7 (Figure 1f), opening the tunnel between TMs 1 and 7 (Figure 1e).

Subsequently, JWH-133 moves to a second energetic minimum in the pathway, located in the bundle-facing part of the CB_2_R tunnel (Figure 4), to mainly interact with Val36^1.35^, Cys40^1.39^, Phe87^2.57^, Phe91^2.61^, His95^2.65^, Ala282^7.36^, and Met286^7.40^ (Figure 2d). We experimentally validated this entrance tunnel by mutating Ala282^7.36^ that is not located at the orthosteric binding site but midway between the lipid- and the bundle-facing part of the CB_2_R tunnel (Figure 3b). It is common in the GPCR field to perform mutagenesis assays to study the influence in affinity or efficacy of amino acids located at the orthosteric binding pocket. Here, we aimed at decreasing the residence time of the ligand by blocking its entrance and, thus, blocking the signaling response (decrease of forskolin-induced cAMP). The Ala282^7.36^Phe mutation, but not Ala282^7.36^ Met, allows a blockade of the effect of JWH-133 because the decrease of forskolin-induced cAMP is of less magnitude in the mutant than in nonmutated receptor (Figure 3b). We propose that a highly stable aromatic cluster between Phe91^2.61^, Phe94^2.64^, His95^2.65^, and Phe282^7.36^ (in the Ala282^7.36^Phe mutation), which cannot be accomplished with Met282^7.36^, blocks the entrance of JWH-133 to the orthosteric binding site. Figure S4 shows a structure-based sequence alignment of the amino acids forming this pathway among the members of class A GPCRs that bind hormone-like signaling molecules derived from lipid species. Finally, JWH-133 moves to the most energetically stable position in the pathway, at the orthosteric binding site (Figure 4).

The pathway of JWH-133 entry to CB_2_R defines two transient binding sites, in addition to the final location at the orthosteric cavity (Figure 4). These transient sites have been proposed to be potential binding sites for synthetic modulators (allosteric modulators or bitopic ligands), becoming important sites for drug discovery due to the high conservation of the traditional orthosteric binding site.^18,19,79^ To analyze this suggestion, we have superimposed our previously reported binding modes of a negative allosteric modulator^63^ and a bitopic ligand^78^ of CB_2_R. Clearly, the bitopic ligand binds at the orthosteric site and the identified transient lipid-facing site. Thus, the lipid-facing site in CB_2_R is analogous to the vestibule or secondary or metastable binding site defined for other GPCRs that bind polar ligands (see Introduction), which is key for ligand desolvation^15,16,40^ and for selectivity,^20,28,77^ but it might not allosterically modulate receptor activity. In contrast, the negative allosteric modulator cannabidiol binds at the identified bundle-facing site, from which it elicits the allosteric modulation.^63^ Thus, the bundle-facing site is an allosteric site in the CB_2_R, located near the orthosteric site, as has been suggested for muscarinic receptors.^79^ In conclusion, our findings have shown that ligand pathway simulations might be a useful tool to identify and delineate cavities for the design of synthetic modulators of GPCRs.

## 5 Data and Software Availability

PACKMOL-Memgen, distributed with AmberTools, is free of charge. MDAnalysis, GROMACS, and PLUMED are open source. FATSLiM and CAVER are licensed under the GNU General Public License. VMD is available to noncommercial users under a distribution-specific license, and PyMOL is commercial software with a paid license.

## Supporting Information Available

Workflow of the computational process, heatmap representation of the cavity opening events, sequence alignment of class A GPCRs that bind hydrophobic-like signaling molecules, and analysis of membrane stability (PDF)
