# Supplementary material for: A Single Point Mutation Blocks the Entrance of Ligands to the Cannabinoid CB2 Receptor via the Lipid Bilayer
Source: J Chem Inf Model. 2022 Oct 27;62(22):5771–9. doi: 10.1021/acs.jcim.2c00865 (PMC9709915; doi:10.1021/acs.jcim.2c00865)
Supplement: Supplementary file 1 — ci2c00865_si_001.pdf [file ci2c00865_si_001.pdf]

Supplementary material for

A single point mutation blocks the entrance of ligands to the cannabinoid CB_2 receptor via the lipid bilayer

Nil Casajuana-Martin[†], Gemma Navarro[§,#], Angel Gonzalez[†], Claudia Llinas del Torrent[†], Marc Gómez-Autet[†], Aleix Quintana García[†], Rafael Franco[⊥,#], and Leonardo Pardo[†,*]

[†]Laboratory of Computational Medicine, Biostatistics Unit, Faculty of Medicine, Universitat Autònoma Barcelona, 08193 Bellaterra (Barcelona)

[§]Department of Biochemistry and Physiology, Faculty of Pharmacy and Food Sciences, Universitat de Barcelona, 08028 Barcelona

[#]Centro de Investigación en Red, Enfermedades Neurodegenerativas (CIBERNED), Instituto de Salud Carlos III, 28031 Madrid

[⊥]Department of Biochemistry and Molecular Biomedicine, Faculty of Biology, Universitat de Barcelona, 08028 Barcelona

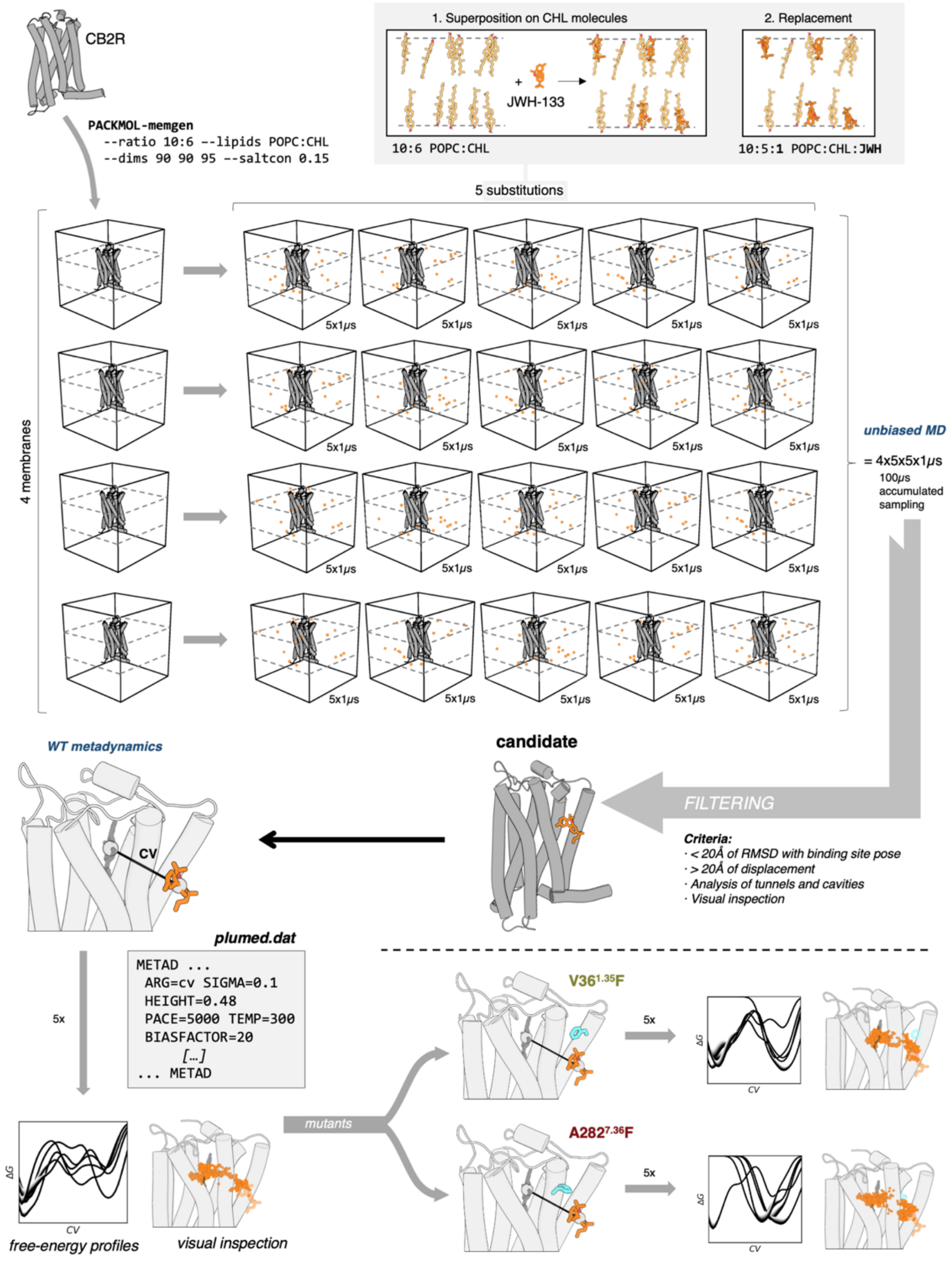

Figure S1. Workflow of the computational process. The inactive structure of CB_2R (PDB id 5ZTY) was given as an input to PACKMOL-memgen to generate four original membrane systems (commands are shown) with a 10:6 POPC:CHL ratio. A random substitution of CHL by JWH-133 (JWH) was performed five times, leading to a 10:5:1 POPC:CHL:JWH ratio. Each of these twenty combinations was subjected to five replicas of unbiased 1µs MD simulation with an aggregate sampling of 100 µs. A filter (rmsd < 20Å relative to the reference docked binding mode + > 20 Å of displacement from the initial position) was applied to the data collected every 10 ns (10,000 snapshots). The structures that spontaneously bound the tunnel between TMs 1 & 7 were selected for WT metadynamics simulations. The CV of choice was the

distance between centers of mass of JWH-133 at the initial (lipid-facing cavity) and final (orthosteric site) points. Five replicas were calculated, using a plumed.dat input file with the depicted settings. Similar simulations were performed for the Val36$^{1.35}$Phe and Ala282$^{7.36}$Phe mutants.

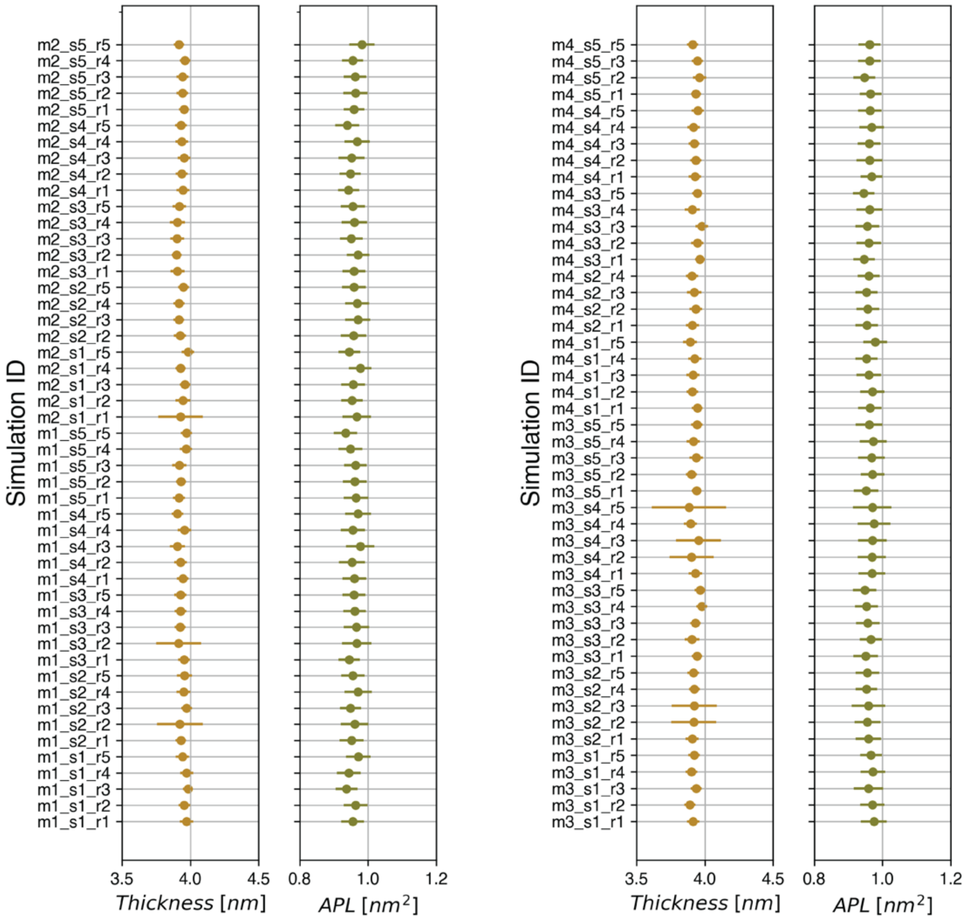

Figure S2. Analysis of membrane stability during the one hundred unbiased 1 μs MD simulations. The lipid bilayer thickness and the area per lipid (APL), calculated with FATSLiM, are shown.

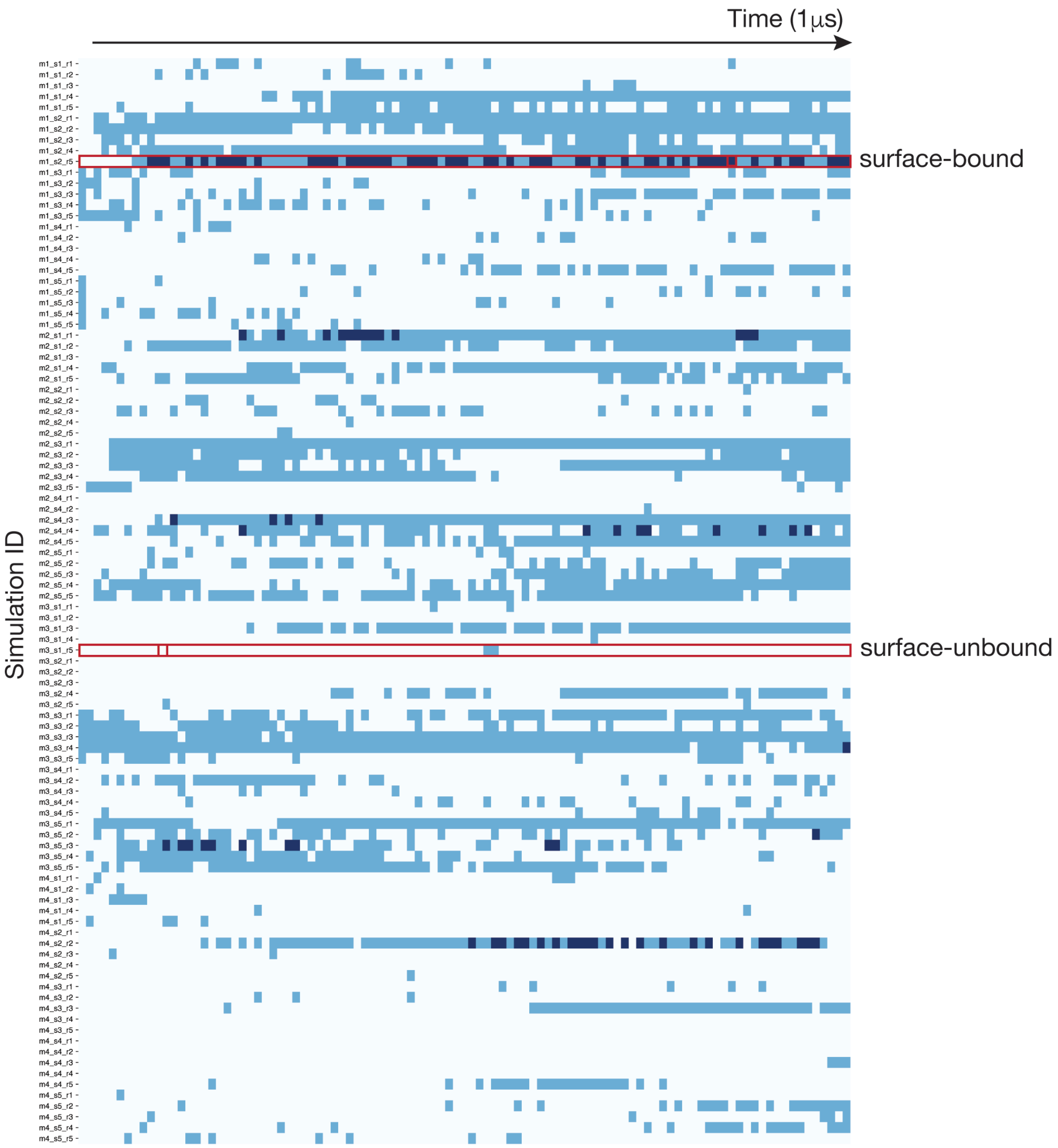

Figure S3. Heatmap representation of the cavity opening events. Snapshots, during the 100 μs of MD simulation, in which Phe283$^{7.37}$ adopts the *trans* conformation or/and the distance between the top of TMs 1 (Cα atom of Thr34$^{1.33}$) and 7 (Cα atom of Val277$^{7.31}$) increases from the initial value of 14.5 Å to values larger than 15.5Å. Light and dark blue indicates whether one or both events are happening, respectively. The trajectory in which the ligand (orange in Figure 1b) spontaneously binds the lipid-facing part of TMs 1 and 7 is highlighted as surface-bound, whereas a trajectory in which these events rarely occur is highlighted as surface-unbound. The selected snapshots for closed and open cavity in Figure 1e are highlighted by a red rectangle.

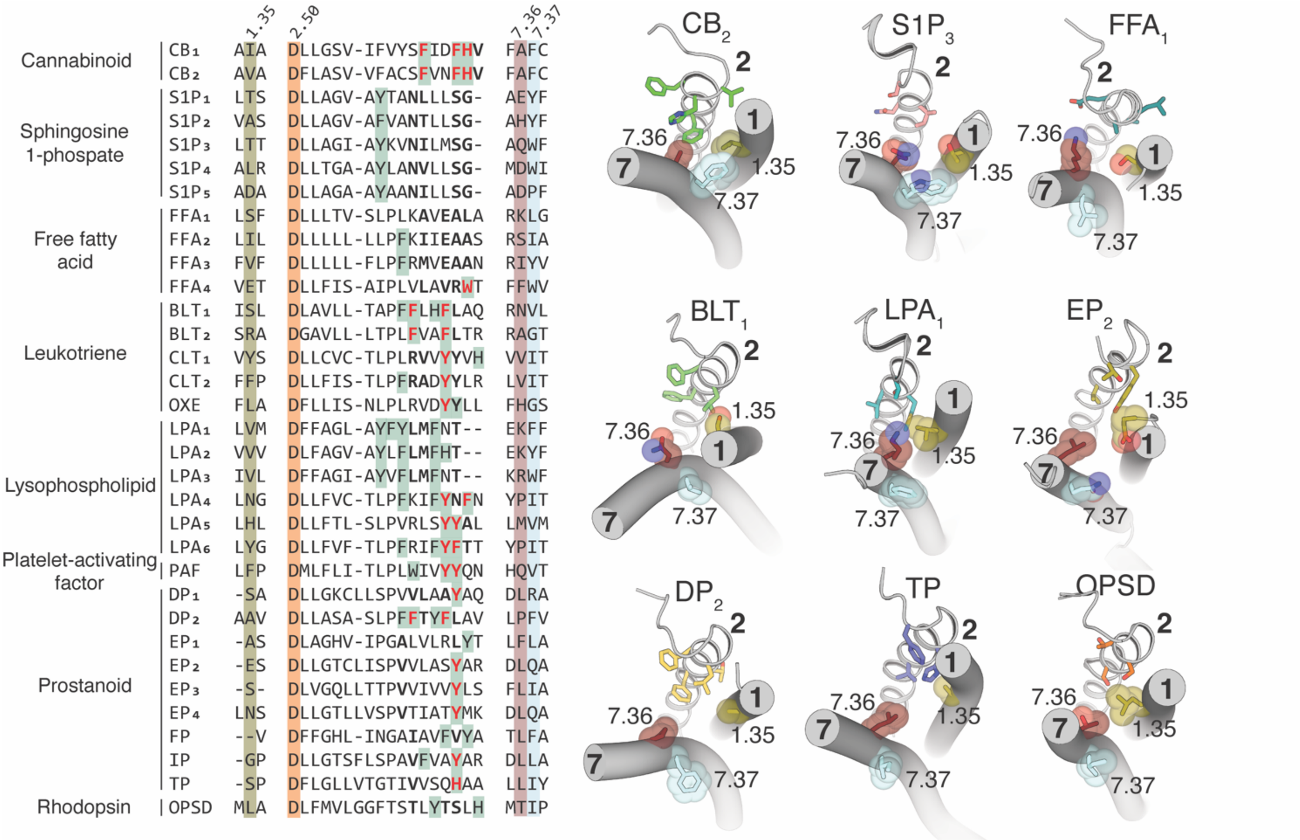

Figure S4. Structure-based sequence alignment of the amino acids forming the pathway of ligand entry to CB$_2$R among the members of class A GPCRs that bind hormone-like signaling molecules derived from lipid species. Sequence alignment of 3 positions of TM 1, 18 positions of TM 2, and 4 positions of TM 7 of the different lipid receptors, grouped by subfamily (left panel). The amino acids involve in the tunnel entrance (1.36 and 7.36) are shown, the amino acids closing/opening the tunnel (7.37) is shown, and aromatic residues in top 10 residues in TM 2 are shown. Residues in TM 2 pointing towards TMs 1 and 7 are highlighted in bold; aromatic residues within this selection are colored in red. Spatial visualization of these residues in different structures (right panels).
